# Decoding ferroptosis: transforming orthopedic disease management

**DOI:** 10.3389/fphar.2024.1509172

**Published:** 2024-12-06

**Authors:** Guanlin Huo, Yumeng Lin, Lusheng Liu, Yuqi He, Yi Qu, Yang Liu, Renhe Zhu, Bo Wang, Qing Gong, Zhongyu Han, Hongbing Yin

**Affiliations:** ^1^ College of Traditional Chinese Medicine, Changchun University of Chinese Medicine, Changchun, China; ^2^ Health Management Center, Nanjing Tongren Hospital, School of Medicine, Southeast University, Nanjing, China; ^3^ Department of Acupuncture and Moxibustion, Shanghai TCM-Integrated Hospital, Shanghai University of Traditional Chinese Medicine, Shanghai, China; ^4^ Department of Blood Transfusion, Lu’an People’s Hospital, The Affiliated Hospital of Anhui Medical University, Lu’an, China; ^5^ Orthopaedic Center, Affiliated Hospital of Changchun University of Chinese Medicine, Changchun, China; ^6^ Department of Orthopaedics, The Eighth Medical Center of PLA General Hospital, Beijing, China; ^7^ Orthopedic Center, The Third Affiliated Hospital of Changchun University of Chinese Medicine, Changchun, China

**Keywords:** lipid peroxidation, antioxidant system, iron metabolism, orthopedic disorders, mechanisms, therapy

## Abstract

As a mechanism of cell death, ferroptosis has gained popularity since 2012. The process is distinguished by iron toxicity and phospholipid accumulation, in contrast to autophagy, apoptosis, and other cell death mechanisms. It is implicated in the advancement of multiple diseases across the body. Researchers currently know that osteosarcoma, osteoporosis, and other orthopedic disorders are caused by NRF2, GPX4, and other ferroptosis star proteins. The effective relief of osteoarthritis symptoms from deterioration has been confirmed by clinical treatment with multiple ferroptosis inhibitors. At the same time, it should be reminded that the mechanisms involved in ferroptosis that regulate orthopedic diseases are not currently understood. In this manuscript, we present the discovery process of ferroptosis, the mechanisms involved in ferroptosis, and the role of ferroptosis in a variety of orthopedic diseases. We expect that this manuscript can provide a new perspective on clinical diagnosis and treatment of related diseases.

## 1 Introduction

Cell death is a crucial mechanism by which organisms maintain homeostasis and equilibrium in their systems. Currently, the predominant focus of research is on programmed cell death (PCD). The content includes autophagy, apoptosis, and various other cell death mechanisms (Fuchs and Steller, 2015; Bertheloot et al., 2021). PCD is controlled by multiple factors, including genetics, signaling pathways, and the cellular environment (Jacobson et al., 1997). These factors influence the development and outcome of systemic immunity, biochemistry, and disease (Su et al., 2015; Tower, 2015). Ferroptosis is distinct from more conventional forms of cell death since it relies on lipid peroxidation and intracellular iron buildup as its primary triggers (Chen F. et al., 2024). The extensive inquiry into PCD has been prompted by these two characteristics. There are noticeable necrotic alterations and morphological differences between ferroptosis and other forms of cell death (Lei et al., 2022). Additionally, the most notable physical features are alterations to the mitochondrial architecture, including a decreased body size, increased density, reduced or absent cristae, and damaged outer membranes (Dixon et al., 2012; Stockwell et al., 2017).

Currently, the majority of studies on ferroptosis mortality mostly investigate its association with cancer, neurological disorders and blood-related conditions (Yan et al., 2021). They are closely associated with lipid metabolism, reactive oxygen species (ROS) and hypoxia, and iron metabolism, all of which involve metabolic pathways related to ferroptosis (Deng et al., 2024; Qiu et al., 2020). Musculoskeletal disorders (MSKs) are the leading cause of long-term disability globally. In some cases, these diseases may progress to the point of causing dysfunction or even death. Presently, numerous studies have been published regarding the impact of ferroptosis on the development and outcome of orthopedic diseases. Therefore, it is essential to consolidate the understanding of the causes and clinical management strategies for iron deficiency in the field of orthopedic diseases.

The notion of ferroptosis was first proposed in 2012 by Dixon et al. (Dixon et al., 2012). In the 1950s, researchers discovered specific changes in the morphology of cystine-deficient cells (Eagle, 1955a; Eagle, 1955b). It was also revealed that the addition of cysteine did not alter the morphological changes of cells triggered by cystine deficiency, which may be mainly due to the difference in intracellular absorption mechanisms between cystine and cysteine (Eagle, 1959; Eagle et al., 1961). Cystine deficiency leads to reduced glutathione and cell death (Bannai et al., 1977). In the liver necrosis model, which is now thought to be caused by ferroptosis, the addition of glutathione and cysteine protects tissues from injury (Mitchell et al., 1973). These studies link cystine, cysteine, and glutathione. By introducing α-tocopherol, an antioxidant, it can be accomplished to reverse cell death caused by cystine deprivation, even without an increase in glutathione levels (Bannai et al., 1977). The findings of this investigation provide further proof that the formation of ROS is a causative component in cell death (Hirschhorn and Stockwell, 2019).

Subsequent investigations have shown that the prevention of such forms of cell death may be achieved by introducing iron chelators and lipophilic antioxidants (Ratan et al., 1994; Ratan et al., 1996; Wang et al., 2004). Glutathione peroxidase 4 (GPX4), discovered in 1982, showed its capacity to suppress iron-catalyzed lipid peroxidation in microsomes. The foremost function of GPX4 is to safeguard phosphatidylcholine-containing liposomes and biofilms against destruction caused by peroxidation (Ursini et al., 1985; Ursini et al., 1982). It is regarded as a crucial enzyme inhibitor that regulates ferroptosis cell death (Arai et al., 1999; Hurst et al., 2001). This protective effect is assumed to be due to GPX4’s ability to shield cells from oxidative stress-induced cell turnover (Yagi et al., 1996). The notion of non-apoptotic types of cell death was first introduced by much research on non-apoptotic caspase-independent cell death, characterized by necrosis-like morphology (Borner and Monney, 1999; Fiers et al., 1999; Loscalzo, 2008).

**FIGURE 1:**
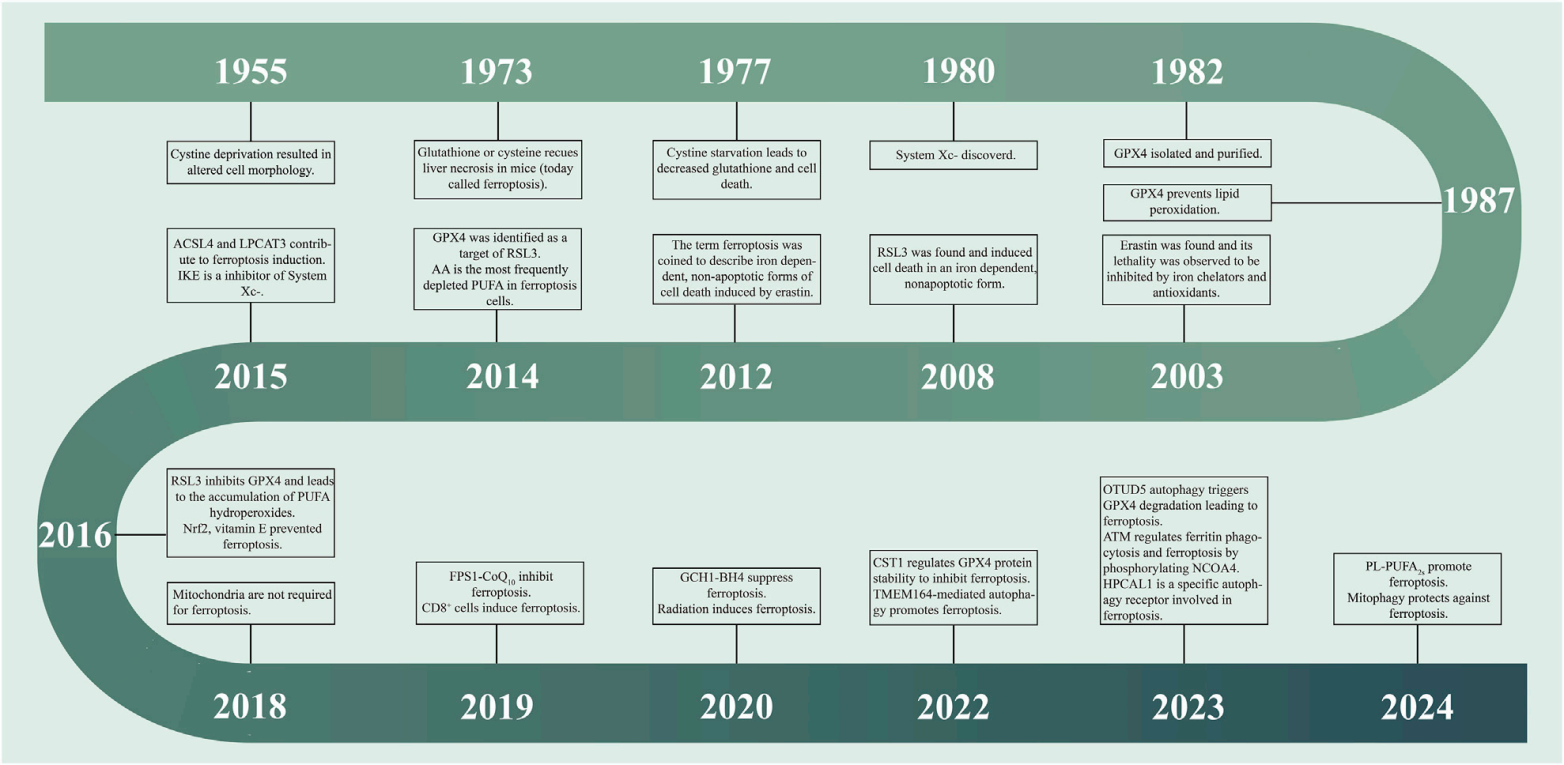
Timeline for development of ferroptosis. Each time point has critical findings on the mechanism of ferroptosis. These findings have led to the refinement of ferroptosis theories as distinct from other modes of ferroptosis. ACSL4: Acyl-CoA synthetase long-chain family member 4; ATM: ataxia telangiectasia mutated; BH4: tetrahydrobiopterin; CD8+: a human leukocyte differentiation antigen; CoQ10: Ubiquinone-10; CST1: Cystatin SN; FSP1: ferroptosis suppressor protein 1; GCH1: Guanosine triphosphate cyclohydrolase-1; GPX4: Glutathione Peroxidase 4; HPCAL1: hippocalcin like 1; LPCAT3: Lysophosphatidylcholine acyltransferase 3; NCOA4: Nuclear Receptor Coactivator 4; NrF2: Nuclear factor erythroid 2-related factor 2; OTUD5:OTU Deubiquitinase 5; PL-PUFA2S: phospholipids with two polyunsaturated fatty acyl tails; PUFA: polyunsaturated fatty acid; RSL3: RAS synthetic lethal 3; System Xc-: A Cystine/Glutamate Reverse Transport System; TMEM164: Transmembrane Protein 164.

Stoxwell’s laboratory conducted a screening of deadly chemicals that specifically targeted cells harboring oncogenic mutations HRAS as big and small T oncoproteins. Due to its evident capacity to eliminate RAS and small T-transformed cells, it was designated as “erastin” (Dolma et al., 2003; Yang and Stockwell, 2008). Nevertheless, no discernible indicators of programmed cell death were seen after the administration of erastin to the tumor cells. Iron chelators and lipophilic antioxidants were successful in preventing the fatal effects of erastin. This finding indicates that erastin has the ability to trigger a kind of cell death that is not associated with apoptosis (Dolma et al., 2003; Yagoda et al., 2007). Another study also discovered that RAS synthetic lethal 3 (RSL3) induces a non-apoptotic form of cell death that is reliant on iron. Subsequent experiments by Dixon and Stockwell confirmed that erastin acts by inhibiting cystine/glutamate reverse transporter (SystemXc-), which reduces the cysteine-dependent synthesis of reduced glutathione (GSH). This is the first explanation of the mechanism of ferroptosis induction. According to these findings, the term ferroptosis was coined in 2012 to describe this iron-dependent, non-apoptotic form of cell death induced by erastin and RSL3 (Dixon et al., 2012; Dixon et al., 2014) (Figure 1).

This review provides an overview of the molecular mechanisms underlying ferroptosis, the connection between ferroptosis and other forms of cell death, the development of ferroptosis in orthopedic diseases, and protocols to target ferroptosis for the treatment of orthopedic diseases. This review aims to enhance the ability of clinicians to provide more accurate guidance in clinical diagnosis and treatment options.

## 2 Ferroptosis mechanism

**FIGURE 2:**
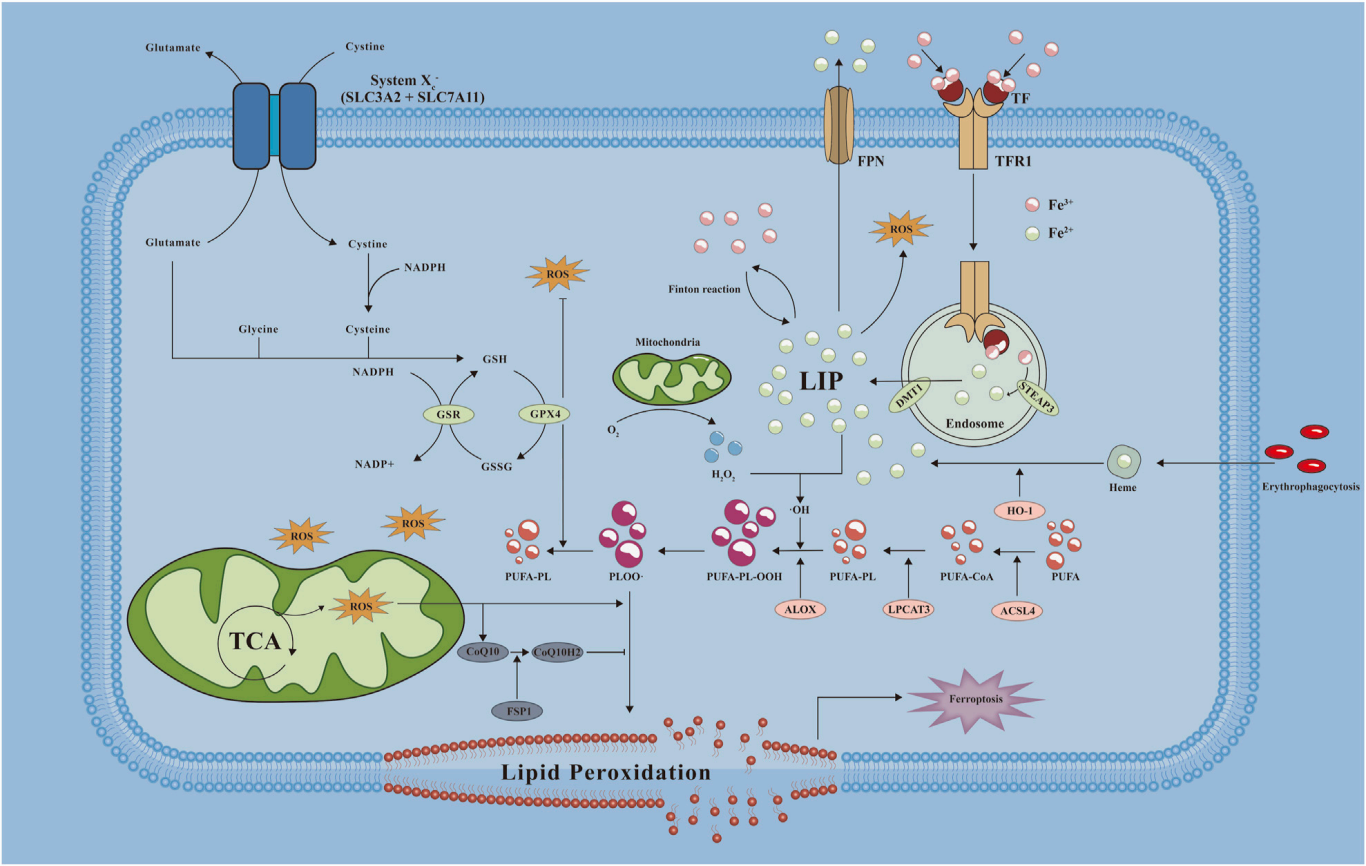
Mechanistic map of ferroptosis occurring in cells. A variety of key molecules, pathways and related reactions are involved in ferroptosis when cells undergo ferroptosis, of which abnormal iron metabolism and lipid peroxidation are key factors in the development of ferroptosis. ·OH: hydroxyl radical; ALOX: Arachidonate Lipoxygenase; CoQ10H2: reduced form of coenzyme Q10; DMT1: divalent metal transporter 1; FPN: ferroportin; GSH: Glutathione, Reduced; GSR: Glutathione-Disulfide Reductase; GSSG: Glutathione, Oxidized; HO-1: Heme oxygenase-1; LIP: labile iron pool; LPCAT3: Lysophosphatidylcholine Acyltransferase 3; NADP+: Nicotinamide adenine dinucleoside phosphate; NADPH: nicotinamide adenine dinucleotide phosphate; PLOO·: Phospholipid hydrogen peroxide radical; PUFA-CoA: polyunsaturated fatty acid- Ubiquinone; PUFA-PL: phospholipids containing polyunsaturated fatty acids; PUFA-PL-OOH: PUFA phospholipid hydroperoxides; ROS: reactive oxygen species; SLC3A2: Solute Carrier Family 3 Member 2; SLC7A11: Solute Carrier Family 7 Member 11; STEAP3: Six-Transmembrane Epithelial Antigen Of Prostate 3; TCA: tricarboxylic acid cycle; TF: transferrin; TFR1: transferrin receptor 1.

Ferroptosis differs from other modes of cell death mainly because of its iron concentration dependence as well as lipid peroxidation. In this process, iron metabolism disorders, antioxidant system imbalance as well as mitochondrial dysfunction are involved in the progression of cellular ferroptosis. We will address in this section the mechanisms by which ferroptosis occurs, including the mode of iron metabolism in organisms, the effect of iron on lipid peroxidation, and the role of the antioxidant system during ferroptosis. And we will introduce how ferroptosis occurs in cells from the perspective of lipid metabolism, energy metabolism, and amino acid transport. We also introduce the perspectives of inflammation, hypoxia and epigenetics to further describe the mechanism of ferroptosis (Figure 2).

### 2.1 Iron metabolism

#### 2.1.1 Systemic iron metabolism

The human body has approximately 3–5 g of iron, which is primarily found in red blood cells and hemoglobin (Wang and Pantopoulos, 2011). The tight management of iron absorption from the meal in the duodenum is a vital mechanism for maintaining homeostasis (Hentze et al., 2010). Mammals experience iron loss by detachment or bleeding from mucosal and skin cells (Chen et al., 2023). Therefore, the regulation of iron levels at the systemic level involves maintaining a balance between iron supply, utilization, and loss (Hentze et al., 2010; Muckenthaler et al., 2017).

Systemic iron metabolism involves the co-participation and co-regulation of multiple pathways. Iron is transported through the blood stream to juvenile erythrocytes and most tissues using transferrin (Tf) (Weichhart, 2024). The reticuloendothelial system can also participate in iron metabolism *in vivo* as an iron reservoir (Comità et al., 2024). Among them, cytochrome b in the duodenum has a strong regulatory effect on iron (Lane et al., 2015). Ferric reductase is coupled to divalent metal transporter 1 (DMT1) or divalent cation transporter (DCT1) to transport Fe^2+^ across the apical membrane oxygenase promotes inorganic iron release from heme in macrophages and enterocytes (Cain and Smith, 2021). Hepcidin binds to ferroportin (FPN), thereby controlling iron transport into the plasma (Wang and Babitt, 2019).

#### 2.1.2 Cellular iron metabolism

Cells possess a comprehensive mechanism for regulating the absorption and metabolism of iron, in comparison to systemic iron metabolism. The plasma membrane exerts strict regulation over the transportation of iron between cells. Cells adhere to transferrin receptor 1 (TfR1) and obtain iron from Tf (Anderson and Vulpe, 2009). Each Tf molecule is able to bind two Fe^3+^ (Chua et al., 2007). The Tf-TfR1 complex is internalized and acidified in endosomes via receptor-mediated endocytosis, facilitating Fe^3+^ release (Dautry-Varsat et al., 1983; Bali et al., 1991). Fe^3+^ is then reduced to Fe^2+^ via six-transmembrane epithelial antigen of the prostate 3 (STEAP3). Fe^2+^ enters the cytoplasm via DMT1 or transient receptor potential protein (TRPML1) (Fleming et al., 1998; Ohgami et al., 2006). The Tf-TfR1 complex then breaks down and enters the next cycle. Non-transferrin bound iron (NTBI) can similarly contribute significantly to cellular iron uptake. When the ability of plasma Tf to bind iron is overloaded, NTBI can rapidly enter the cell to replace the function of Tf (Craven et al., 1987; Wright et al., 1986). The labile iron pool (LIP) refers to a collection of iron that has a low molecular weight and is weakly bound to chelating agents. Iron that is taken up through Ff-dependent or independent pathways enters the labile LIP (Kruszewski, 2003). Furthermore, in situations where cellular iron levels are insufficient, iron regulatory protein 1 and iron regulatory protein 2 attach to stem-loop structures known as iron response elements (IREs) in messenger RNAs that encode various proteins involved in iron metabolism (Meneghini, 1997; West and Oates, 2008; Nemeth et al., 2004).

#### 2.1.3 Iron overload

Iron overload is a condition that occurs due to various factors. Iron overload is typically categorized as either primary or secondary (Cherukuri et al., 2005). The main component of secondary iron load is the iron that builds up because of transfusions used to treat disorders of the red blood cell system (Larrick and Hyman, 1984). β-thalassemia can involve both increased absorption of iron and an excess of iron from transfusions (Anderson, 2007). Primary iron overload is characterized by the increased absorption of excessive iron due to the enhanced reabsorption function of the small intestine. Diseases related typically exhibit low levels of hepcidin in the bloodstream in relation to the amount of iron in the body (Uchida et al., 1983; Hentze et al., 2004).

#### 2.1.4 Iron toxicity

When the binding capacity of plasma Tf is excessive, the free iron concentration in plasma increases, NTBI increases rapidly (Bernstein, 1987; Trenor et al., 2000). The result of this process is the gradual accumulation of iron in these organs, which leads to toxicity (Dickinson and Connor, 1994; Beutler et al., 2000; Asada-Senju et al., 2002). The formation of highly ROS is initiated by labile cellular iron (Craven et al., 1987). ROS are injurious, nevertheless cells possess multiple defense mechanisms to minimize ROS accumulation (Finch, 1994). When the body’s defense mechanisms reach max capacity, they can result in cell destruction. Lipid peroxidation is caused by ROS attacking cell and organelle membranes (Craven et al., 1987).

### 2.2 Lipid peroxidation

#### 2.2.1 Mechanisms of lipid peroxidation

Ferroptosis characterized by elevated levels of lipid peroxidation and a lack of ability to remove lipid peroxides (Dixon et al., 2012; Stockwell, 2022; Yang et al., 2016). Because phospholipids (PL) in mammalian cell membranes contain large amounts of PUFAs, PLs are the major sites of lipid peroxidation (Li et al., 2024a). Lipid peroxidation is categorized into three distinct stages: initiation, propagation, and termination (Yan et al., 2021). The initiation process removes hydrogen atoms from allylic carbons in PUFAs by ROS, RNS, and RLS, forming lipid radicals. Hydroxyl and hydroperoxyl radicals are generated through the Fenton reaction involving ferrous iron and hydrogen peroxide. Lipid peroxidation can be initiated by reactive nitrogen species (RNS) like peroxynitrite, reacting with nitric oxide and superoxide. Lipid radicals undergo oxygen-to-peroxyl reactions, producing lipid peroxides that can be converted by GPX4 or broken down into reactive lipid species (RLS), such as 4-HNE and MDA, perpetuating lipid peroxidation and triggering cellular signaling.

#### 2.2.2 Phospholipid peroxidation

Phospholipid peroxidation, reliant on iron, leads to membrane degradation and cell death (Liang et al., 2022). PL’s two fatty acyl chains (sn-1 and sn-2) contribute to its chemical diversity, with sn-1 having SFA or MUFA, and sn-2 having SFA, MUFA, or PUFA (Harayama and Riezman, 2018). PUFA-PLs are the substrate for PL peroxides. They can be converted to PLOOH enzymatically or non-enzymatically. Normally, GPX4 reduces PLOOH to phosphatidyl alcohols, terminating the Fenton reaction (Ursini et al., 1982; Seiler et al., 2008).

PUFA-PLs are more prone to ferroptosis due to their susceptibility as substrates for PL peroxidation (Doll et al., 2017; Kagan et al., 2017). PL may undergo peroxidation through non-enzymatic reactions, facilitated by metals like iron. Intracellular iron can become labile and react with H2O2, initiating the Fenton reaction which converts Fe^2+^ and Fe^3+^ ions and H2O2 into reactive radicals and lipid peroxidation.

If neutralization is slow, PLOOH can engage in an iron-catalyzed Fenton reaction, forming lipid radicals, which can spread oxidation to nearby PUFA-PL molecules (Chen et al., 2020; Geng et al., 2018; Gao et al., 2016; Hou et al., 2016). Iron-dependent enzymatic reactions, like those catalyzed by lipoxygenases (LOXs), which possess non-heme iron, play a crucial role in the peroxidation of phospholipid membranes, influencing ferroptosis regulation (Liang et al., 2022; Kuhn et al., 2015).

15-LOX facilitates conversion of PE and plays a role in peroxidizing membrane phospholipids (Erba et al., 2024). This conversion is very selective and specific, resulting in the catalysis of arachidonoyl (AA) and adrenoyl (AdA) groups (Stoyanovsky et al., 2019). GPX4 is crucial to lipid peroxidation. GPX4 converts poisonous PLOOH to harmless PLOH (Ursini et al., 1982). Phospholipids/neutral lipid hydroperoxides are reduced to their hydroxyl derivatives by GPX4 (Björnstedt et al., 1995; Imai, 2004; Imai and Nakagawa, 2003). nhibiting GPX4 activity promotes PL peroxidation, PLOOH buildup, and ferroptosis (Friedmann Angeli et al., 2014; Yang et al., 2014).

#### 2.2.3 The role of ROS in lipid peroxidation

As indicated, ROS have significance to several cell death processes. ROS greatly impact lipid peroxidation, which is the hallmark of ferroptosis. DNA, protein and lipid can react with ROS (Halliwell and J, 2022). Lipid peroxidation occurs when oxygen free radicals attack PUFAs, resulting in the generation of lipid free radicals. These radicals then proceed to target neighboring PUFAs and membrane proteins, ultimately leading to membrane lipid peroxidation (Yang et al., 2016; Liang et al., 2022; Que et al., 2018). ROS generated via the Finton reaction catalyzed by iron (Lei et al., 2019; Toyokuni et al., 2017). ROS-PUFA reactions cause lipid peroxidation. Oxidative stress triggered by ROS in lipids leads to cell damage (Rochette et al., 2022).

### 2.3 System X_c_^−^/GSH/GPX4

SystemXc-is a transporter that is located on the cell membrane. The transportation and exchange of cystine can be facilitated by System Xc-, which also allows for the transport of glutamate (Fantone et al., 2024). Cystine enters cells and is reduced to cysteine, which combines with glutamate and glycine to form GSH. System Xc-controls GSH production through disulfide bonds between solute carrier family 7 member 11 (SLC7A11) and solute carrier family 3 member 2 (SLC3A2) (Liu et al., 2020a; Jiang and Sun, 2024). SLC7A11 facilitates cystine to glutamate transfer in a 1:1 ratio (Fantone et al., 2024). Cysteine is subsequently reduced to cysteine by thioredoxin reductase 1 (TrxR1) (Conrad and Sato, 2012; Mandal et al., 2010). SLC3A2 plays a crucial role in regulating the transport function of SLC7A11 and ensuring its stability (Nakamura et al., 1999).

Glutamate, cysteine, and glycine form GSH, with glutamate-cysteine synthase and glutathione synthase as catalysts for its production (Bansal and Simon, 2018; Forman et al., 2009). Within the cell, most of the space is occupied by reduced glutathione (Diaz-Vivancos et al., 2015). Glutathione disulfide (GSSG) is the most common oxidized form of glutathione. Both reduced and oxidized forms of glutathione are capable of undergoing interconversion (Niu et al., 2021). The progression of ferroptosis is contingent upon the levels of GSH((Guo et al., 2021)). GSH is an antioxidant that neutralizes ROS, inhibits lipid peroxides, and maintains cellular redox equilibrium by adjusting NADP/NADPH and GSH/GSSG ratios (Diaz-Vivancos et al., 2015; Sahoo et al., 2022; Zhong et al., 2022). GSH acts as a cofactor for GPX4, preventing lipid peroxidation and ferroptosis by converting LOOH into LOH (Jia et al., 2020).

Cysteine in GPX’s protein superfamily redox residues catalyzes redox processes (Flohé et al., 2022; Xie et al., 2023). GPX4 can prevent lipid oxidation and biofilm destruction by using GSH’s reducing equivalent (Ursini et al., 1982; Nishida et al., 2022). GPX4 reduces intracellular peroxides, especially phospholipid hydroperoxides (Ursini et al., 1982; Thomas et al., 1990). GPX4 is most typically expressed in the cytosol, followed by mitochondrial and nuclear classes. Apoptosis resistance is mostly due to mitochondrial GPX4 (Liang et al., 2009). GPX4 is one of the main regulators performing ferroptosis. Gpx4 safeguards mitochondria from peroxide damage (Liu et al., 2024). GPX4 loss or malfunction causes intracellular peroxide buildup and ferroptosis (Xie et al., 2023). GSH functions as a crucial cofactor for GPX4, inhibiting lipid peroxidation and ferroptosis through the reduction of LOOH to LOH (Li FJ. et al., 2022).

### 2.4 Glucose metabolism and ferroptosis

Glucose serves as a primary energy source, transformed into pyruvate through glycolysis, then entering the tricarboxylic acid (TCA) cycle and oxidative phosphorylation (OXPHOS) (Liu J. et al., 2021; Nie et al., 2020). During anoxia, the enzyme lactate dehydrogenase catalyzes the conversion of pyruvate into lactate (Sonveaux et al., 2008). Glucose 6-phosphate can also produce NADPH and biosynthetic precursors through the pentose phosphate pathway (PPP) (Lee et al., 2017; Martinez-Outschoorn et al., 2017). NADPH plays a crucial role in the transformation of oxidized glutathione into reduced glutathione (Dong et al., 2015; Liu et al., 2019). Mitochondria play a crucial role in energy metabolism and are responsible for generating ROS (Ashton et al., 2018; Zong et al., 2016). The occurrence of ferroptosis is closely linked to energy metabolism.

#### 2.4.1 Glucose-dependent energy metabolism

Energy metabolism involves electron transfer for glucose-dependent systems, with electrons transferred to oxygen, generating ROS (Chen and Zweier, 2014; Corbet et al., 2016; Förstermann et al., 2017). Blocking TCA cycle in tumor cells disrupts electron transfer, reducing ROS levels and preventing ferroptosis (Gao et al., 2015; Gao et al., 2019a). Ferroptosis is linked to TCA cycle intermediates and enzymes. Glutamine relates to iron-induced apoptosis, while glutamine synthase 2 facilitates iron-mediated programmed cell death (Suzuki et al., 2022). α-Ketoglutarate, a TCA cycle intermediate, induces iron-dependent cell death like glutamine. Citrate synthase and acyl-CoA synthesis family member 2 control the production of fatty acids and impact the process of lipid peroxidation (Gao et al., 2019a). Pyruvate dehydrogenase kinase 4 prevents cell death caused by iron by inhibiting pyruvate dehydrogenase, while malic enzyme 1 deficiency in synovial sarcoma tumors makes cells vulnerable to ferroptosis (Song et al., 2021). Deficiency of malic enzyme 1 in tumor models of synovial sarcoma renders cells vulnerable to ferroptosis (Brashears et al., 2022). Fumarate hydratase inactivation causes cell death in hereditary leiomyomatosis and renal cell cancer, linked to GPX4 inactivation (Kerins et al., 2018). Mutations occur in isocitrate dehydrogenases (IDH1 and IDH2) in cancer cells (Gbyli et al., 2022; Varn et al., 2022). Based on this premise, cancer cells are susceptible to erastin-induced ferroptosis (Kim H. et al., 2020). IDH1 can additionally suppress GPX4 expression and facilitate GSH depletion, thereby triggering ferroptosis (Wang et al., 2019).

#### 2.4.2 Ferroptosis in PPP

PPP is an energy metabolism branch that produces NADPH, essential for fatty acid synthesis and preventing ferroptosis by converting GSSG to GSH. Ferroptosis inducers decrease NADPH activity (Shimada et al., 2016). NADPH facilitates cystine uptake through SLC7A11, converting it to cysteine for GSH generation (Shu et al., 2020; Liu X. et al., 2021). Thioredoxin (Trx) helps trigger ferroptosis by being a redox pathway. Blocking Trx’s activity triggers ferroptosis. NADPH helps convert oxidized Trx to its reduced form (Sun et al., 2001). NADPH can also enzymatically convert coenzyme Q10 (CoQ10) into CoQ10-H2, resulting in inhibiting lipid peroxidation (Bersuker et al., 2019; Doll et al., 2019). Ferroptosis triggers PPP-NADPH production, boosting GSH, Trx, and CoQ10-H2 antioxidant effects.

### 2.5 Inflammation and ferroptosis

Iron deficiency is linked to inflammation, triggering production of inflammatory molecules that stimulate lipid peroxidation (Chen X. et al., 2021; Chen Y. et al., 2022; Sun et al., 2020). Ferroptosis is linked to inflammatory signaling pathways. Triggering ferroptosis with erastin or RSL3 activates the JAK-STAT pathway through IFN-γ in tumor cells (Barrat et al., 2019; Kong et al., 2021). The JAK2-STAT3 signaling pathway positively correlates with hepcidin expression, influencing systemic iron metabolism through regulation (Yang L. et al., 2020; Ren et al., 2021; Kowdley et al., 2021).

The NF-κB pathway is associated with the activation of inflammation and the innate immune response (Hoesel and Schmid, 2013). Ferroptosis is mediated by the NF-κB signaling pathway, which is activated by ferroptosis inducer RSL3, regulating System X_c_^−^ transmission and interacting with heme oxygenase 1 to metabolize heme and ferrous iron (Pulkkinen et al., 2011; Ryter, 2021). NF-κB signaling promotes ferritin heavy chain 1(FTH1) expression in response to TNF-α (Kou et al., 2013). Ferritin light chain expression (FTL) increases in lipopolysaccharide (LPS)-stimulated macrophages, limiting NF-κB signaling and reducing TNF-α, IL-1β, and inflammation (Zarjou et al., 2019).

Inflammasomes have a strong correlation with lipid peroxidation. NLRP3 inflammasomes upregulated in response to GPX4 inhibition, linked to lipid peroxidation (Xie et al., 2022). Inhibition of NLRP3 reverses oxidative stress in a lipid peroxidation model (Orecchioni et al., 2022). Suppressing NLRP3 led to increased GPX4, elevated GSH levels, and reduced phospholipid peroxides (Meihe et al., 2021). The cGAS-STING pathway interacts with ferroptosis, inducing oxidative stress and STING translocation. STING inhibition decreases ferroptosis sensitivity (Li C. et al., 2021). Ferroptosis from high iron diet and GPX4 deficiency can cause pancreatic cancer in mouse models, affecting cGAS-STING signaling (Dai et al., 2020).

The MAPK pathway triggers ferroptosis through inflammatory activation, inducing pro-inflammatory cytokines, suppressing GPX4 and System X_c_^−^, and leading to neuroinflammation and cell death (Zhu et al., 2021). Excessive iron activates ERK1/2 and p38, causing oxidative stress and peroxide formation through the MAPK pathway (Salama and Kabel, 2020; Ikeda et al., 2019). Application of antioxidants resulted in the inhibition of the MAPK pathway and a reduction in peroxide concentration (Fu et al., 2018; Cavdar et al., 2020).

### 2.6 Hypoxia and ferroptosis

Hypoxia is a physiological reaction that occurs due to various internal or external conditions (Tano and Gollasch, 2014; Chen G. et al., 2022; McClelland and Scott, 2019). Within the hypoxic environment, some distinct signaling pathways are triggered, mostly through the mediation of hypoxia-inducible factor (HIF) (Schito and Semenza, 2016; Kaelin and Ratcliffe, 2008). Oxidative stress increases under hypoxia and ROS accumulation is the main cause of ferroptosis (Honda et al., 2019). The HIFs family has three isoforms: HIF-1, HIF-2, and HIF-3. HIF-1 forms in extreme oxygen deprivation, while HIF-2 forms in moderate oxygen deprivation (Koh and Powis, 2012; Cowman and Koh, 2022).

The HIFs family has three isoforms: HIF-1, HIF-2, and HIF-3. HIF-1 forms in extreme oxygen deprivation, while HIF-2 forms in moderate oxygen deprivation (Zheng X. et al., 2023). The inhibiting impact of this substance affects both normal cells and malignant cells (Kumar and Choi, 2015; Pan et al., 2021). HIF-1α prevents ferroptosis by stabilizing SLC7A11 and activating hypoxia response elements that control glutathione metabolism (Hu et al., 2022; Lin Z. et al., 2022). HIF-2α acts as a positive regulator of ferroptosis (Johansson et al., 2017; Singhal et al., 2021). HIF-2α triggers ferroptosis by activating genes like ACSL4 Cigarette smoke exposure boosts HIF-2α, leading to myotube apoptosis (Zhang L. et al., 2022). Further investigation is needed to investigate the diametrically opposite effects of HIF-1α and HIF-2α.

### 2.7 Antioxidation mechanism

#### 2.7.1 Vitamin E (α-tocopherol)

α-tocopherol is a free radical-trapping antioxidant that blocks peroxidation propagation and inhibits phospholipid peroxide accumulation (Burton and Ingold, 1981; Traber and Atkinson, 2007; Ingold and Pratt, 2014). By competitively binding PUFAs and scavenging hydroxyl radicals, α-tocopherol has been demonstrated to block the action of LOX, hence preventing ferroptosis (Feng and Stockwell, 2018; Angeli et al., 2017). *In vitro* studies have shown thatα-tocopherol can protect GPX4 knockout mice from ferroptosis (Kagan et al., 2017; Friedmann Angeli et al., 2014). α-tocopherol can synergize with GPX4 to inhibit lipid peroxidation (Carlson et al., 2016; Wortmann et al., 2013).

#### 2.7.2 NRF2

NRF2 is a transcription factor that controls cellular antioxidant reactions and the effects of oxidative stress (Kajarabille and Latunde-Dada, 2019). Reducing the expression of NRF2 enhances vulnerability to ferroptosis (Xie et al., 2016). NRF2 additionally governs SLC7A11 (Rosell et al., 2023). NRF2 regulates iron/ferroheme metabolism at the metabolic level and governs the activity of FTL and FTH1, which are crucial proteins involved in iron metabolism (Agyeman et al., 2012; Harada et al., 2011; Kerins and Ooi, 2018). NRF2 is also involved in regulating NADPH, GSH and GPX4 synthesis (Jiang et al., 2024; Luchkova et al., 2024).

### 2.8 Epigenetic

Epigenetics is a dynamic process that includes DNA methylation, histone modification, and non-coding RNA (ncRNA) regulation, which allows gene expression to change without changing the DNA sequence (Wang N. et al., 2023; Cavalli and Heard, 2019). In addition to being impacted by traditional signaling pathways, ferroptosis-related genes are also controlled by epigenetic processes.

#### 2.8.1 Histone modifications

Gene expression is determined by the structure of the chromosome. Through acetylation, methylation, and ubiquitination, histone modification controls the structure of DNA (Yang M. et al., 2020). It is regulated by the bromodomain-containing protein (BRD) family, histone acetyltransferases (HATs), and histone deacetylases (HDACs) (Sabari et al., 2017). BRD4 suppression or inhibitors led to ferroptosis in tumor cells, suppressing GPX4 and System X_c_^−^ expression (Sui et al., 2019). Histone acetylation stimulates transcription, while deacetylation inhibits transcription (Barneda-Zahonero and Parra, 2012). NRF2 recruits P300/CBP-associated factor (PCAF), which regulates ferroptosis through H3K9ac levels, controlling SLC7A11 expression (Chung et al., 2019). HDACs inhibit epithelial-mesenchymal transition (EMT) markers’ expression in cancer cells, resulting in ferroptosis initiation (Liu L. et al., 2021; Lee J. et al., 2020). Histone methylation regulates transcription by modifying H3 and H4 histone N-terminal lysine/arginine residues (Yi et al., 2017a; Yi et al., 2017b). Histone methyltransferases are enzymes that add methyl groups to specific sites on histone proteins, including H3K4 and H3K9 (Li et al., 2019). He elevated expression of GPX4 in cancerous cells could be attributed to the augmented abundance of H3K4me3 in its promoter region (Ma M. et al., 2022). Increasing H3K4me3 abundance upregulates Acyl-CoA Synthetase, suppressing ferroptosis (Zhang et al., 2020). The expression of SLC7A11 is tied to histone 2A ubiquitination and histone 2B ubiquitination (Ling et al., 2022; Horniblow et al., 2022).

#### 2.8.2 DNA methylation

DNA methylation plays a crucial role in the ferroptosis process as it regulates the synthesis of PUFAs and levels of ROS (Jiang et al., 2017; Lee JY. et al., 2020). DNA methylation represses gene activity related to lipid peroxidation, preventing ferroptosis, and inactivates GPX4 promoter causing cellular ferroptosis (Zhang J. et al., 2022; Ling et al., 2022). Methylation is controlled by ferroptosis, which occurs in iron-rich environments and affects NRF2(213).

#### 2.8.3 Noncoding RNAs

MiRNAs regulate epigenetic inheritance by binding to 3′UTRs and interfering with mRNA translation (Zhang and Liu, 2021). Research has shown that miRNAs target proteins that regulate iron metabolism, leading to ferroptosis (Li X. et al., 2021; Wei et al., 2021). In addition, miRNAs can also inhibit System X_c_^−^ and GPX4 expression to promote ferroptosis (Fan et al., 2021; Xu et al., 2020; Deng et al., 2021; Yadav et al., 2021; Ding et al., 2020). Researchers focus on Circular RNAs (circRNAs), which counteract miRNA inhibition on GPX4 expression through endogenous competition (Xu et al., 2020; Chen W. et al., 2021). CircRNA can also act as a sponge for miRNA, resulting in the upregulation of SLC7A11 expression (Wu et al., 2021). Long non-coding RNAs (lncRNAs) can directly interact with the p53 gene and trigger ferroptosis via the p53-GPX4 axis (Chen C. et al., 2021). Additionally, lncRNAs can indirectly facilitate ferroptosis by promoting apoptosis (Wang Z. et al., 2021).

Multiple mechanisms are involved in the epigenetic regulation of ferroptosis, as stated in conclusion. In addition, there are inextricable connections between its systems, which are consistent areas that require additional research.

## 3 Ferroptosis and other cell death pathways

### 3.1 Ferroptosis and autophagy

Lysosomes are attached to autolysosomes during autophagy in order to facilitate cellular turnover and metabolism. The breakdown of internal metabolic activities during autophagy results in the creation of autophagosomes (Luo and Tao, 2020; Zhou et al., 2020). There is growing evidence that ferroptosis requires autophagy’s involvement (Mizushima and Levine, 2020). T Ferroptosis-inducing medications can cause GPX4 degradation by autophagy, which is subsequently mediated by the enzyme acid sphingomyelinase, which is essential for the metabolism of sphingolipids (Thayyullathil et al., 2021). Autophagy causes the amount of free iron in the bodies of mice with subarachnoid hemorrhage to increase, which leads to ferroptosis of free iron in their bodies grow, which ultimately results in ferroptosis (Patil et al., 2021). A few results that are comparable to one another demonstrate that autophagy increases ferritin degradation (Hou et al., 2016; Liu et al., 2020b; Tian et al., 2020; Ma et al., 2017).

### 3.2 Ferroptosis and pyroptosis

Pyroptosis is an inflammatory form of cell death that relies on the involvement of caspases (Chen et al., 2019). It is characterized by a similar pattern to ferroptosis, involving membrane damage, accompanied by ROS accumulation and iron dependence (Xu R. et al., 2021). ROS, via iron-dependent activation, facilitate caspase-related pathways, leading to the degradation of ferritin and the initiation of pyroptosis (Zhou et al., 2018).

### 3.3 Ferroptosis and cuproptosis

Cuproptosis is characterized by an aberrant metabolism of copper ions. High levels of copper ions can lead to protein toxicity, which can ultimately result in the death of cells (Tsvetkov et al., 2022). Mitochondria link ferroptosis with cuproptosis. Mitochondria play a significant role in the production of ROS and directly contribute to cell death caused by iron. Additionally, in the mitochondrial TCA cycle, the process of glutaminolysis, which leads to a shortage of cysteine, is also responsible for cell death caused by iron (Gao et al., 2019b). Observations revealed morphological alterations in mitochondria affected by cuproptosis, with mitochondrial respiration and acting a significant part in this process (Tang et al., 2022). Furthermore, GSH serves as a central point linking iron toxicity with copper toxicity. A study has proven that the drugs sorafenib and erastin, which are ferroptosis inducers, increase cell death in primary hepatocellular carcinoma cells when combined with a copper ionophore (Wang W. et al., 2023). This effect is achieved by decreasing the synthesis of GSH. GSH also forms a complex with copper to decrease the buildup of copper within cells (Liu and Chen, 2024).

### 3.4 Ferroptosis and apoptosis

Apoptosis in programmed cell death is one of the most intensively studied. Initiation of apoptosis activates caspase (Yuan and Ofengeim, 2024). Caspases can cause cells to form apoptotic typical morphology, such as nuclear lysis and rounding of the cell shape (Rajagopalan et al., 2024). Ferroptosis was discovered as a novel programmed cell death independent of apoptosis because it caused cell death without caspase activation and could not be reversed by caspase inhibitors (Wu P. et al., 2023). But recent studies have found an inextricable link between iron death and apoptosis. For example, p53 apoptosis stimulating protein inhibitor (iASPP) inhibits p53-induced apoptosis, while iASPP also plays an anti-ROS role, thereby promoting Nrf2 accumulation and nuclear metastasis (Li et al., 2020). And Nrf2 is a protective mechanism against iron death. Perez et al. found that iron death is mutually exclusive with apoptosis (Perez et al., 2020). In addition, erastin induced p53 to promote apoptosis in A549 lung cancer cells (Huang et al., 2018). Erastin can also induce oxidative stress and cause caspase-9-dependent cell death (Huo et al., 2016).

## 4 Ferroptosis and orthopedic disease

**FIGURE 3:**
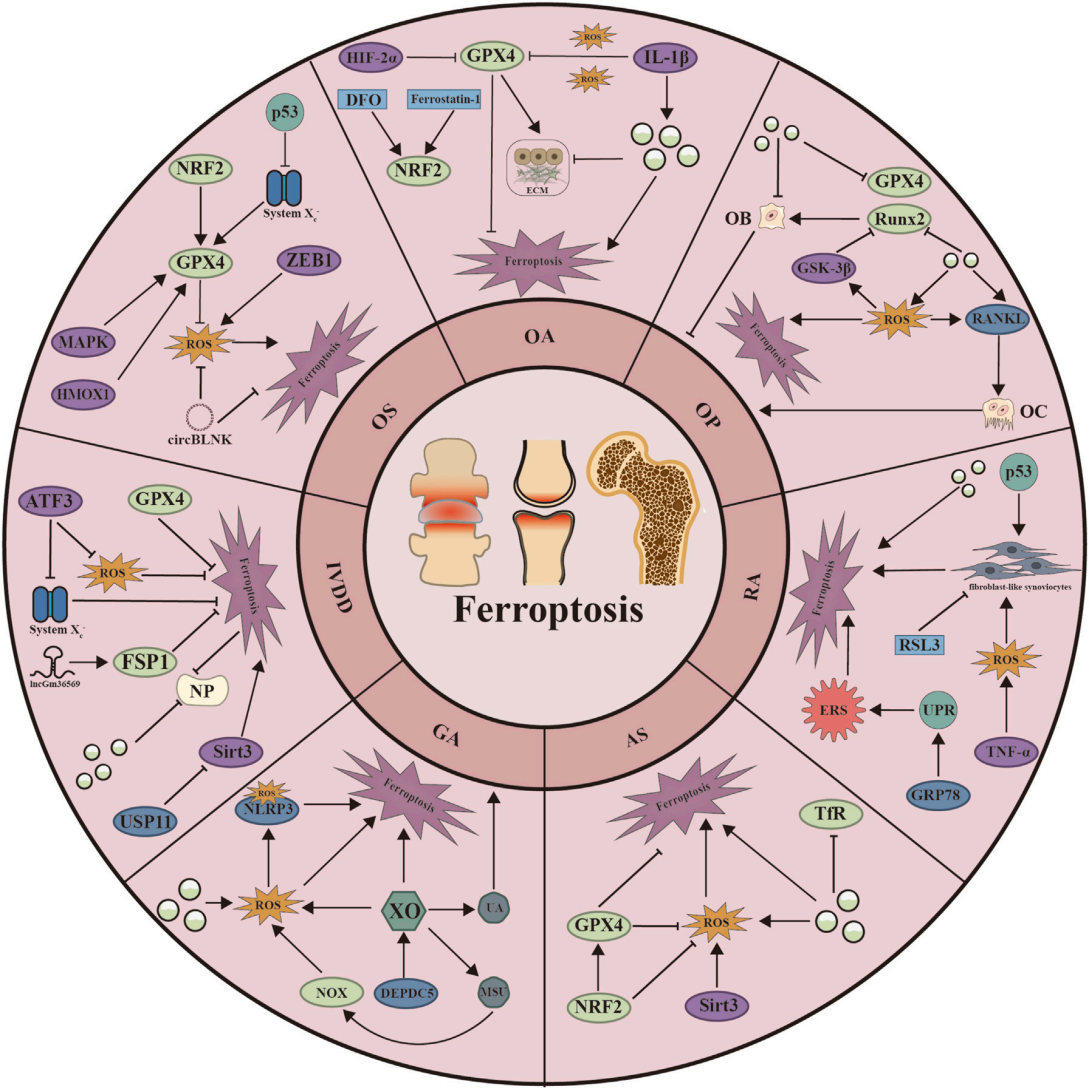
Schematic representation of the mechanisms by which ferroptosis regulates seven skeletal diseases. ATF3: Activating Transcription Factor 3; CircBLNK: circular RNA; DEPDC5: DEP Domain Containing 5; DFO: Deferoxamine; ECM: Extracellular matrix; ERS: Endoplasmic Reticulum Stress; GRP78: Glucose regulatory protein 78; GSK-3β: Glycogen Synthase Kinase 3 Beta; Hif-2α: Hypoxia Inducible Factor 2 Subunit Alpha; HMOX1: Heme Oxygenase 1; IL-1β: Interleukin 1 Beta; MAPK: Mitogen-Activated Protein Kinase; MSU: monosodium urate; NLRP3: NLR Family Pyrin Domain Containing 3; NOX: nitrogen oxides; NP: Nucleus pulposus; OB: Osteoblast; OC: osteoclast; RANKL: Receptor Activator of Nuclear Factor-κB Ligand; Runx2: RUNX Family Transcription Factor 3; Sirt3: Sirtuin 3; TNF-α: Tumor Necrosis Factor Alpha; UA: urine acid; UPR: unfolded protein response; USP11: Ubiquitin Specific Peptidase 11; XO: xanthine oxidase; ZEB1: Zinc Finger E-Box Binding Homeobox 1.

The relationship between ferroptosis and disease is a hot research direction in recent years. The development of skeletal system disorders has also been widely documented to be strongly associated with iron death. In addition, multiple regulatory mechanisms of ferroptosis also play a critical role in the progression of skeletal system diseases. Therefore, we will discuss how ferroptosis affects the occurrence and outcome of bone diseases in this section, and try to enrich clinical treatment strategies through the exploration of the mechanism of iron death in seven bone diseases (Figure 3).

### 4.1 Iron metabolism in bone and cartilage

Chondrocytes are the only cells that constitute articular cartilage and act to be responsible for the metabolism of the extracellular matrix (ECM) (Pettenuzzo et al., 2023). Studies have demonstrated that chondrocytes, which are affected by inflammation, tend to accumulate ROS and have modified expression of ferroptosis-related proteins in models of OA. More precisely, the expression of GPX4 and SLC7A11 in chondrocytes was reduced when inflammation was triggered by IL-1β and by creating a simulated iron overload environment. Utilizing ferroptosis inhibitors resulted in a decrease in ROS levels and a reduction in cytotoxicity (Yao et al., 2021). Another study revealed that inflammatory stimuli can disturb the iron equilibrium in chondrocytes. The expression of TfR1 was upregulated while the expression of FPN was downregulated in chondrocytes after treatment with IL-1β and TNF-α. An excess amount of iron in cartilage leads to the destruction of cartilage, resulting in oxidative stress and damage to the mitochondria (Jing et al., 2021).

### 4.2 OA

From the aforementioned investigations, it is evident that there is a strong correlation between the occurrence of OA, characterized mostly by cartilage destruction, and iron depletion. Oa is a closely related disease to age and a disease that is easily disabling in chronic diseases. Inflammation, cartilage degeneration, and synovial hyperplasia can be seen in OA. Abnormal iron metabolism can directly contribute to OA by causing inflammation, in addition to the indirect damage to cartilage that is commonly associated with the condition (Burton et al., 2020). Elevated ferritin levels are one of the risk factors for OA. Imaging analysis showed a positive correlation between ferritin levels and the severity of arthritis (Cai et al., 2021; Kennish et al., 2014). One element affecting older patients’ morbidity is gender. Research has shown that OA affects older women on more occasions than older males, and that this difference is related to postmenopausal estrogen levels (Ko and Kim, 2020). Nonetheless, ferritin levels are negatively connected with estrogen levels, and iron levels in the serum of postmenopausal women are 200%–300% higher than those of non-menopausal women, according to current research. Because of this distinction, postmenopausal women experience a much higher prevalence than do men (Park et al., 2012; Zhang et al., 2001; Ke et al., 2021). There is a substantial correlation between blood problems and OA, according to a Mendelian randomization study (Xu J. et al., 2022). Hemophiliac individuals have an increase in red blood cells in the joint space because of continuous methemoglobin release and prolonged bleeding, which causes iron accumulation and ferroptosis (van Vulpen et al., 2018; Bhat et al., 2015). Patients with hemochromatosis who have OA have also been observed to accumulate iron in the joint space; dysmorphic cartilage has been restored after excess iron has been removed (Richette et al., 2010; Heiland et al., 2010).

The mechanism of OA resulting from ferroptosis involves inhibition of antioxidant pathways. In OA, inhibition of GPX4 and SLC3A2 expression has been shown (Liu H. et al., 2022; Miao et al., 2022). ECM degrades when GPX4 expression is suppressed (Miao et al., 2022). Type II collagen (collagen II) was also expressed in ECM by ferroptosis inhibitors, and this process was reversed after ferroptosis inhibitor treatment (Yao et al., 2021). Activation of Nrf2 can also prevent ferroptosis (Xu C. et al., 2022). In addition, HIF-2α stimulates lipid peroxidation and suppresses GPX4 and SLC7A11 to facilitate chondrocyte ferroptosis (Zhou et al., 2021). Piezo1, a mechanosensitive ion channel, is also involved in iron metabolism in OA by inhibiting the GSH-GPX4 axis (Wang S. et al., 2022; Ma et al., 2021). Overall, ferroptosis in OA involves multiple aspects. Among them, inflammation and antioxidant inhibition are the main causes of OA.

### 4.3 AS

AS is a rheumatic bone disease caused by inflammation. The location of AS is centered on the sacroiliac joint and affects the surrounding joints (Thomas and Brown, 2010; Rostom et al., 2010). The main symptom of AS is inflammatory spinal pain, accompanied by bone erosion and ligamentous osteophytes (Tam et al., 2010). Although ferroptosis caused by lipid peroxidation is rarely reported in AS, it is not difficult to find from the study of specific serum proteomics and metabolome student markers in AS patients that the main reasons affecting ferroptosis in AS are iron levels and oxidative stress response. Studies have reported that serum TfR1 levels are low in AS patients, while platelet iron content remains high (Fischer et al., 2012; Feltelius et al., 1986). Decreased antioxidant capacity is one of the characteristics of AS. A study on serum oxidation and antioxidation in AS patients highlighted decreased antioxidant capacity and increased oxidative stress index in AS patients (Karakoc et al., 2007). AS patients with metabolic syndrome are more susceptible to oxidative stress (Pishgahi et al., 2020). GPX, which is closely associated with ferroptosis, was found to have decreased expression in mouse models of AS (Feng et al., 2020; Dong, 2018).

### 4.4 GA

GA is characterized by hyperuricemia and urate deposition (Zhang Y. et al., 2022). Abnormal iron metabolism and antioxidant imbalance are one of the pathogeneses of GA. A Mendelian randomization study revealed a relationship between ferritin and risk of gout. This study is the first to demonstrate a positive association between serum ferritin and the risk and frequency of gout (Fatima et al., 2018). In a study on the association between markers of iron status and the risk of hyperuricemia in Chinese adults, the researchers found a positive correlation between serum ferritin, transferrin and hyperuricemia (Li et al., 2018). The same results were found in another US National Health and Nutrition Examination Survey (Ghio et al., 2005). Maintaining close to iron deficiency levels has also been demonstrated as a protective factor in GA (Facchini, 2003). Xanthine oxidase (XO) is the only source of urate. XO enhances its activity when combined with iron (Jomova et al., 2024; Maiti et al., 2022). A direct relationship between iron and uric acid is therefore concluded.

### 4.5 RA

The autoimmune illness RA is typified by progressive bone damage and synovial hyperplasia. Teratogenicity in RA is caused by pannus development, chronic inflammation, and bone loss. Recent research has demonstrated that characteristics unique to ferroptosis can also be seen in RA, and these discoveries have established a connection between RA and ferroptosis. Treatment for RA may be improved by having a better understanding of the general process of ferroptosis in RA.

Abnormal iron metabolism is the first element leading to ferroptosis in RA. A Mendelian randomization study of genetic data from a large genome-wide association study of 257,953 individuals suggests that individuals with genes associated with higher iron levels may have a lower risk of RA (Wu, 2024). Another Mendelian randomization trial yielded similar results, showing a negative correlation between iron intake and RA (Wang et al., 2024a). According to a clinical investigation, RA patients had considerably lower serum iron levels and higher serum TFR values (Stefanova et al., 2018). Peripheral blood iron levels are lower in patients with severe RA (Wu et al., 2022).

Another important factor in the progression of RA is ROS, which has been shown to rise approximately fivefold in the mitochondria from whole blood and monocytes of RA patients. The main manifestations of ROS include increased oxidative stress and decreased antioxidant levels (Ferreira et al., 2021). Oxidative stress occurs when there is an imbalance between ROS production and the body‘s antioxidant defenses. In RA, elevated ROS levels were observed in synovial fluid, blood, and affected joint tissues. These ROS contribute to joint inflammation and injury by promoting activation of pro-inflammatory signaling pathways, inducing cytokine production, and enhancing proliferation of fibroblast-like synoviocytes (FLS). The resulting inflammatory and oxidative damage leads to degeneration of cartilage and bone, which are hallmark features of RA (Mueller et al., 2021). The immune system plays a crucial role in RA, where T cells, B cells, and macrophages contribute to a chronic inflammatory state. Oxidative stress can affect the function and survival of these immune cells. For example, ROS can modulate T cell activation and differentiation, skewing immune responses toward more inflammatory features (Hassan et al., 2011). High levels of inflammation trigger lipid peroxidation, and excess ROS also generate pannus at home, and such circulatory effects accelerate the progression of RA (Phull et al., 2018; Zhou et al., 2012). During RA, macrophages release a significant number of inflammatory factors. TNF-α has the most intricate role among them all. One the one hand, as previously mentioned, TNF-α can increase inflammation by inducing ROS generation through NADPH oxidase (Latchoumycandane et al., 2012). TNF-α has been observed to stimulate GSH production and cystine absorption, however. Long-term TNF exposure can prevent NADPH Oxidase (NOX) from producing ROS, shielding FLS from ferroptosis (Wu et al., 2022).

The endoplasmic reticulum (ER) is the major organelle responsible for protein synthesis and lipid metabolism in eukaryotic cells. ER stress (ERS) refers to a series of pathological conditions such as overload of folding mechanisms and disruption of redox balance that occur during protein synthesis in the ER (Tabas and Ron, 2011). The unfolded protein response (UPR) is a compensatory response initiated in the ERS state (Walter and Ron, 2011). Evidence suggests that in the setting of RA, inflammation provokes ERS and proteins are synthesized via UPR (Park et al., 2014). ERS shares signaling pathways with ferroptosis, which makes the role of ERS have to be considered when studying ferroptosis in RA (Li C. et al., 2023). Glucose-regulated protein 78 (GRP78) promotes aberrant protein breakdown under UPR in the ERS state (Grootjans et al., 2016). GRP78-specific antibodies were identified in 63% of RA patients (Bläss et al., 2001). ERS and increased GRP78 expression can be brought on by ferroptosis inducers. P53 functions as a tumor suppressor gene in ferroptosis and ERS. It has been discovered that p53, which induces cell cycle arrest to promote chondrocyte death, is substantially expressed in RA patients (Ghosh et al., 2012; Takatori et al., 2014; Hong et al., 2017).

### 4.6 OP

The skeleton of the human body is always in a state of dynamic balance. Osteoblasts (OBs) and osteoclasts (OCs) are key players in this balance and occupy approximately 90% of the skeletal composition (Cui et al., 2022). Different cells are used to differentiate OBs from OCs. Bone marrow-derived mesenchymal stem cells (BMSCs) are the source of OBs, which is a component of bone production. Originating from the monocyte/macrophage lineage of hematopoietic cells, OCs is the main factor for bone resorption and is abundant in mitochondria and lysosomes (Boyce, 2013; Kim JM. et al., 2020; Sommerfeldt and Rubin, 2001). An imbalance between the production and resorption of bone results in OP, a metabolic bone disease that increases the risk of fractures. Ferroptosis is implicated in the pathophysiology of OP, according to numerous studies (Yang et al., 2022).

BMSCs serve as precursors for the generation of OBs (Lin et al., 2019). Runt-related transcription factor 2 (Runx2) regulates BMSCs to promote differentiation into OBs under normal physiological conditions. Other transcription factors that regulate differentiation into OBs include alkaline phosphatase (ALP) and osteocalcin (OCN) (Komori, 2022). The accumulation of body iron leads to an increase in ferritin expression in BMSCs, which inhibits transcription factors crucial for osteoblast development (Balogh et al., 2016). Similar ones have now been observed in a series of experiments: ferroptosis downregulates the OBs phenotype and promotes OBs death (Xu P. et al., 2022; Ma et al., 2020); inhibition of PI3K-Akt-mTOR prevented ferroptosis in BMSCs and upregulated Runx2 and ALP expression (Lan et al., 2022a); and several closely related pathways, including GPX4 and Nrf2, can regulate ferroptosis in OBs (Messer et al., 2009). The OBs ferroptosis process is also influenced by other gene expressions. In an *in vitro* investigation, iron mortality was noted in OBs treated with ferric ammonium citrate, and the genes TfR1 and DMT1, which are in charge of cellular iron uptake, were discovered to be overexpressed (Luo et al., 2022). In MC3T3-E1 cells, excess iron ions also increased the expression of the apoptosis gene and NOX4 (Tian et al., 2016).

OCs differentiate under the influence of activating the receptor activator of the nuclear factor-κB (RANK)-RANK ligand (RANKL) pathway. RANK-RANKL is a specific representation of OCs and reflects OCs number and activity. Research indicates that increased RANKL expression during iron overload conditions stimulates the development of OCs and ultimately results in OP (Yang J. et al., 2020; Ma J. et al., 2022). RANKL-induced differentiation of OCs involves ferroptosis, and the mechanism by which RANKL-induced ferroptosis in OCs is mediated is ferritin autophagy. For OCs to survive, intracellular iron levels are consequently essential. In addition, this study discovered that HIF-1α can effectively prevent osteopenia-related osteopenia by blocking ferritin autophagy (Ni et al., 2021).

Hematological diseases are also one of the causes of OP involved in ferroptosis. For example, people with hemochromatosis are more likely to have osteopenia, and some of them even develop OP, which is closely related to iron accumulation (Baschant et al., 2022). Osteoporotic fractures are more common in thalassemia patients when their bodies’ ability to excrete iron is compromised by prolonged, frequent blood transfusions and iron buildup. Studies of a similar nature have verified a positive correlation between the frequency of blood transfusions and the risk of fracture (Ekbote et al., 2021; Dede et al., 2016).

There are two types of OPs: primary and secondary. Primary OP mostly refers to senile OP and postmenopausal osteoporosis (PMOP), while secondary OP is primarily Diabetic osteoporosis (DOP) and glucocorticoid osteoporosis (GIOP). Abnormal glucose and lipid metabolism may be the pathogenesis of ferroptosis in OBs in DOP (Chen et al., 2023). The researchers discovered that reduced GPX4 expression in DOP bone tissue in mice led to cellular ferroptosis. It was also verified that turning on the Nrf2/Heme Oxygenase-1 (HO-1) pathway may undo this outcome (Ma et al., 2020). Osteocyte mortality in DOP was successfully prevented by focusing on ferroptosis or HO-1, which broke the vicious cycle between HO-1 activation and lipid peroxidation (Yang et al., 2022). Mitochondrial ferritin (FtMt) is generally considered a tool for mitochondria to regulate free iron content. In the DOP model, high expression of FtMt reduced iron-induced lipid peroxidation, whereas its expression is known to instead cause mitophagy in OBs (Wang et al., 2022b). Increased serum ferritin and decreased GPX4 expression were also observed in the mouse DOP model. Therefore, high glucose environment not only inhibits osteoblast expression, exacerbates trabecular degeneration, osteopenia, but also activates ferroptosis-related gene expression and inhibits the antioxidant system (Lin Y. et al., 2022).

The pathogenesis of PMOP is closely related to estrogen. Iron plays an important role in PMOP. In a study of 728 postmenopausal women, iron was found to be an important risk factor for the onset of PMOP (Okyay et al., 2013). Postmenopausal bone loss might be addressed by taking dietary iron supplements within reasonable bounds (Wylenzek et al., 2024). HIF-1α specific inhibitors have also been found to prevent bone loss in ovariectomy (OVX). Continuous steroid hormone administration impairs osteoblast differentiation activity and lowers antioxidant system capacity, which results in GIOP (Yang et al., 2021a). Patients receiving long-term steroid therapy are more likely to have trabecular bone destruction and osteoporotic fractures. Decreased expression activity of GPX4 and the System X_c_^−^ was found in the high-dose dexamethasone-induced GIOP model (Lu et al., 2019).

Based on these findings, we conclude that there is a close relationship between OP pathogenesis and iron metabolism, and the mechanism also involves lipid peroxidation and oxidative stress. Modulation of osteoblast and osteoclast ferroptosis is a potential treatment option for OP.

### 4.7 OS

Osteosarcoma (OS) is a malignant bone tumor arising from mesenchymal cells and is mostly primary (Yu and Yao, 2024). OS can lead to persistent joint pain, limited mobility, and even susceptibility to lung metastases (Zhang et al., 2018). Epidemiological adjustment reveals that the incidence of OS is closely related to age and ethnicity. Primary OS is more common in men and occurs in the long bones of the lower extremities, with large variations in incidence across ethnic groups (Mirabello et al., 2009). In addition, the age of onset tends to be more in adolescents, which may be the result of rapid skeletal growth during adolescence, and OS is therefore more likely to occur at the ends of long bones in adolescents (Mirabello et al., 2011).

Studies have shown that ferroptosis can inhibit OS progression and reduce OS chemoresistance. The status of the System X_c_^−^-GSH-GPX4 axis is critical in OS progression. By inhibiting the demethylation of H3K9me3 at the SLC7A11 promoter region in the System X_c_^−^, the ability of the System X_c_^−^ to prevent ferroptosis can be reduced (Chen M. et al., 2021). Osteosarcoma cells are resistant to ferroptosis because of a decrease in P53, one of the tumor suppressor genes. P53 binding to SLC7A11 is inhibited and is the main cause (Wang and Pan, 2023). Zinc finger structure E-box-binding homeobox 1 (ZEB1) is involved in lipid metabolism *in vivo*. Overexpressed ZEB1 leads to ROS accumulation (Liu et al., 2023). In transcriptomic experiments, mitochondria from the knockdown ZEB1 group showed ferroptosis-like changes and were involved in the ferroptosis process in OS (Jiacong et al., 2023).

NcRNAs are also involved in the process of ferroptosis. It was shown that genes inhibiting ferroptosis were repressed following miR-206 overexpression in OS cell lines, whereas genes promoting ferroptosis were increased than expression (Li L. et al., 2023). MiR-188-3p targets GPX4 and its expression is reduced in OS tissues, which contributes directly to ferroptosis in OS (Li Z. et al., 2023). LncRNAs can modulate OS resistance and resist OS metastasis (Argenziano et al., 2021). Despite the fact that circRNAs development and cognition are still in their infancy, there is evidence linking circRNAs to tumor metastasis and progression (Zhang et al., 2019). It has been shown that circBLNK and GPX4 are significantly upregulated in OS tissues, promote OS progression, and avoid OS cell ferroptosis (Li Z. et al., 2023).

### 4.8 IVDD

As a degenerative disease, IVDD is the main cause of cervical and low back pain, and about 80% of low back pain is related to IVDD (Li et al., 2024b). Nucleus pulposus (NP) and annulus fibrosus (AF) are the main components of the intervertebral disc and are the main responsible for intervertebral disc function (Le Maitre et al., 2015). Although IVDD is an age-related degenerative disease, current studies suggest that ferroptosis plays an important role in it.

Ferroptosis is involved in IVDD through multiple pathways. The first is to affect normal physiological function by interfering with iron metabolism. Patients’ disc tissue showed reduced expression of FTH (Yang et al., 2021b). Additionally, there was an iron-dose dependent degeneration of cartilage endplates (Wang W. et al., 2022). When FPN is dysfunctional in IVDD, intracellular iron is excessive, which aggravates ferroptosis-induced IVDD (Lu et al., 2021). Some small molecule compounds, such as amino acids, enzymes, and transcription factors, which target the regulation of lipid metabolism and anti-oxidation are also involved in the process of ferroptosis in IVDD. Homocysteine (Hcy), derived from methionine and cysteine, is an important substance in cellular physiology (Mudd et al., 1985). Hcy can cause many musculoskeletal diseases through cellular ferroptosis (Koh et al., 2006; Fayfman et al., 2009). Epidemiological investigation, hyperhomocysteinemia is an important risk factor for IVDD. Inhibition of GPX4 methylation prevented Hcy-guided oxidative stress and ferroptosis (Fan et al., 2023). Activating transcription factor 3 (ATF3) is a member of the ATF/CREB family of transcription factors responsible for regulating signaling pathways and cellular metabolism (Rohini et al., 2018). It has now been demonstrated that ATF3 is a positive regulator of ferroptosis (Wang et al., 2020). In tumor cells, ATF3 can inhibit System X_c_^−^ expression, and inhibit GPX4 and induce ferroptosis (Wang Y. et al., 2021). Results of a bioinformatics experiment revealed that ATF3 gene differences were located in the fourth place in the hub ferroptosis gene ranking in the spinal cord injury model (Gupta et al., 2023). And clinical observations have also found that ATF3 is highly expressed in IVDD, and the mechanism is through the inhibition of SLC7A11 and SOD2 (Li Y. et al., 2022).

Angiogenesis of vascularized granulation tissue is a major feature of IVDD, and much neovascularization is also responsible for keyboard tissue degeneration. Angiogenic vascularized granulation tissue is a major feature of IVDD, and many new vessels are found in NP and are also responsible for disc tissue degeneration (Xiao et al., 2020). As such, hemoglobin numbers were significantly higher in NP than in other surrounding tissues. When IVDD occurs, the iron content in NP is too high, which is the main cause of aggravated ferroptosis (Shan et al., 2021). Notably, HO-1 has a dual regulatory role in ferroptosis. As an antioxidant, it can inhibit ferroptosis, but at the same time, it is characterized by iron concentration dependence, which activates ferroptosis at high iron content (Fang et al., 2019; Adedoyin et al., 2018). A simultaneous increase in HO-1 and iron accumulation was found in the rat IVDD model (Zhang et al., 2021), and clinical studies have also confirmed increased HO-1 expression in NP(358). Such results are strongly associated with neovascularization. As one of the regulatory heme transcription factors, BTB Domain And CNC Homolog 1 (BACH1) expression is closely associated with IVDD. *In vivo* experiments confirmed that knockdown of BACH1 could increase GPX4 and SLC7A11 expression in IVDD, thereby inhibiting ferroptosis (Yao et al., 2023). Sirtuin 3 (Sirt3) is a critical regulator of ROS. High expression of ubiquitin-specific protease 11 (USP11) alleviates ferroptosis caused by oxidative stress. Sirt3 has been found to increase oxidative stress and induce ferroptosis to promote IVDD, while USP11 can bind to Sirt3 and stabilize Sirt3 to slow IVDD progression (Zhu et al., 2023a).

Epigenetics also regulates ferroptosis in IVDD. MiR-10A-5p mediated overexpression of IL-6R in disc cartilage is responsible for ferroptosis in IVDD (Bin et al., 2021). Circ0072464 downregulation and miR-431 upregulation were observed in IVDD, and this result triggered high NRF2 expression, thereby promoting NP proliferation to alleviate IVDD and improve prognosis (Yu et al., 2022). MiR-672-3p promotes ferroptosis during spinal cord injury by downregulating ferroptosis suppressor protein 1 (FSP1) (Wang F. et al., 2022). As a competitive RNA for miR-5627-5p, lncGm36569 induced upregulation of FSP1 to alleviate ferroptosis.

## 5 Targeted therapies in bone diseases affected by ferroptosis

According to the characteristics of ferroptosis and its mechanism of action in bone diseases, it is necessary to find suitable and efficient treatment options for bone diseases caused by ferroptosis. According to the previous discussion, it is known that antioxidant disorders and iron metabolism disorders are the underlying mechanisms of ferroptosis, so therapeutic regimens targeting these two aspects are worthy of in-depth study. Currently relevant treatment options have been reported several times, but an overview of their overall is lacking. Therefore, we try to summarize the current treatment ideas through the content of this part, hoping to help the experimental and clinical application.

### 5.1 Treatment strategies for OA

Cartilage damage is a cardinal feature of OA. As mentioned above, cartilage damage is caused by nothing more than abnormal ROS production and iron metabolism. Regulation of iron metabolism can affect chondrocyte survival. Iron chelators are drugs that are effective against diseases caused by iron accumulation. Iron load is a marker of ferroptosis, so rational use of iron chelators is an effective treatment to cope with ferroptosis. For clinical application, three iron chelators—Deferoxamine (DFO), deferasirox (DFX), and deferiprone (DFP)—have been approved (Mobarra et al., 2016). DFO is an iron chelator approved by the US Food and Drug Administration. DFO was shown to prevent IL-1β-induced upregulation of matrix metallopeptidase 13 (MMP13), and this result also indirectly confirmed that iron is involved in chondrocyte apoptosis in OA (Jing et al., 2020). DFO can also reverse MMP13 activation triggered by erastin, an ferroptosis inducer, and reduce protection against chondrocyte injury by promoting NRF2 pathway activation (Guo et al., 2022). In addition, after DFO treatment, cartilage under hypoxia showed higher ultimate tensile strength and pyridinoline (a collagen protein of mature articular cartilage), although the aim of this finding was to evaluate the mechanical properties of new cartilage, it can also be seen from the results that regulating iron content has far-reaching significance for cartilage tissue (Otarola et al., 2022). In a mouse model, it was found that by intra-articular injection of ferrostatin-1 (an ferroptosis inhibitor), the NRF2 system is activated, attenuating IL-1β-induced ROS accumulation and relieving chondrocyte breakdown, which is a new way to treat OA (250). Another animal experiment also demonstrated that mitochondrial morphology was restored in chondrocytes undergoing ferroptosis after joint injection of ferrostatin-1 and astaxanthin, and collagen II was upregulated due to attenuation of IL-1β (Wang et al., 2022e). Hypoxic environment impacts the body in a complex state, as mentioned above. Several recent studies have focused on the key role of HIF in OA progression (Gonzalez et al., 2018). D-mannose, an isomer of glucose, has been reported to inhibit LPS-induced IL-1β production, which is considered an effective treatment for OA (Torretta et al., 2020). Recent studies have confirmed that D-mannose can inhibit HIF-1α-mediated ferroptosis in chondrocytes (Zhou et al., 2012). HIF-2α is also a non-negligible factor in OA progression. Relevant studies have confirmed that HIF-2α can lead to cartilage destruction by affecting the expression of genes responsible for metabolism in chondrocytes (Saito et al., 2010; Yang et al., 2010; Yang et al., 2015). D-mannose can inhibit HIF-2α to reduce the sensitivity of chondrocytes to ferroptosis (Zhou et al., 2021). Moreover, D-mannose inhibited OA degeneration brought on by IL-1β in rat chondrocytes by triggering autophagy via the AMPK pathway (Lin Z. et al., 2021). Some medicinal ingredients from traditional Chinese medicine are also widely used to treat OA. Icariin, the main component of herb *Epimedium*, has been demonstrated to reduce the expression of IL-1β, MMP, and GRP78, and its mechanism of action is to activate the System X_c_^−^/GPX4 pathway to inhibit ferroptosis (Luo and Zhang, 2021; Pan et al., 2017). Stigmasterol, the main component of *Achyranthes bidentata*, also acts on IL-1β to reduce its chondrocyte damage and regulates ferroptosis through sterol regulatory elements combined with transcription factor 2 (Mo et al., 2021). Intra-articular injection of Cardamonin, one of the extracts of ginger, also inhibited IL-1β-mediated cartilage explanation and regulated ferroptosis through the p53 pathway (Gong et al., 2023).

### 5.2 Treatment strategies for AS

There are few reports on the treatment of AS in ferroptosis, but a number of studies have dominated the prediction of ferroptosis genes closely related to AS. With these predicted genes as primary target points, therapeutic strategies for AS can be identified. Li et al. constructed a protein network of ferroptosis and AS and collected gene expression profiles of AS patients through the GEO database, and concluded that DNA Damage Inducible Transcript 3 and Heat Shock Protein Family B (Small) Member 1 are target genes for inducing ferroptosis in AS cells after enrichment analysis (Li Q. et al., 2022). Another analysis identified Small Ubiquitin Like Modifier 2 and NADH:Ubiquinone Oxidoreductase Subunit S4 as hub genes for ferroptosis in AS cells by constructing differential gene and protein networks (Rong et al., 2022). Dong et al. screened Chloride Intracellular Channel 4 and Tripartite Motif Containing 21 (TRIM21) as key genes in ferroptosis-regulated AS by using a disease prediction model for differential genes involved in cell death, with TRIM21 expression elevated in male patients (Dong et al., 2024). It has been established that acrylamide raises the risk of AS. Several cancers are caused by aflatoxin, which is produced when food is heated. Additionally, it can raise the risk of AS by causing autophagy-dependent ferroptosis. It is therefore advised to limit the sources of acrylamide in meals (Wang H. et al., 2023).

### 5.3 Treatment strategies for GA

Inflammation and ROS are key factors in the pathogenesis of GA and the mechanism of ferroptosis. Targeting ROS-NLRP3 for GA is the primary strategy. Multiple ROS-NLRP3 blockers have been demonstrated to treat GA (Zhang et al., 2024). Multiple natural medicines have proven effective in treating GA. Carvacrol blocked ROS-NLRP3-mediated inflammation, decreased oxidative stress, and decreased uric acid levels in GA patients (Riaz et al., 2022). The natural flavonoid compound rutin was demonstrated to inhibit ROS production and inhibit ROS-NLRP3 inflammatory activation thereby improving joint swelling in a quail GA model (Wu H. et al., 2023). The non-coding RNA lncRNA ZNF883 is one of the key genes identified as ferroptosis leading to GA (Shao et al., 2024). Targeting lncRNA Zinc Finger Protein 883 to treat GA is therefore a drug worth investigating. Targeted modulation of XO is another way. DEP Domain Containing 5 (DEPDC5) subunit deficiency can lead to increased XO and ROS accumulation, which leads to ferroptosis (Li S. et al., 2024). Targeting DEPDC5 pharmaceuticals may be one way to treat GA. Drugs observed from models in which non-ferroptosis leads to GA also decrease XO levels, suggesting that these are potential agents for the treatment of GA. *Artemisia argyi* essential oil could decrease XO and upregulate GPX4 in the hepatic ferroptosis model induced by bisphenol A (Cui et al., 2023). Empagliflozin, a selective inhibitor of sodium-glucose cotransporter 2, alleviated doxorubicin-induced myocardial ferroptosis and decreased XO expression (Quagliariello et al., 2021). The results of transcriptomic and metabolomic analysis showed that exposure to PM2. Five environment reduced antioxidant capacity, increased XO expression, and ultimately led to ferroptosis in mice. Avoiding PM2. Five is therefore also one of the ways to prevent GA resulting from ferroptosis (Shi et al., 2022).

### 5.4 Treatment strategies for RA

Inhibition of synovial hyperplasia to restore synovial homeostasis is an effective treatment for RA (Sandhu and Thelma, 2022). Antioxidant dysregulation due to FLS is a risk factor for RA. Promoting ferroptosis in FLS has therefore emerged as a way to treat RA. Imidazolone erastin (IKE) and the TNF antagonist etanercept induced ferroptosis in FLS and reduced RA symptoms (Wu et al., 2022). Glycine can promote FLS ferroptosis through S-adenosylmethionine mediated methylation of the GPX4 promoter (Ling et al., 2022). Asiatic acid can die from its FLS iron by increasing Fe^2+^ (Sun et al., 2024). Quercetin is a natural flavonoid, and cells treated by quercetin not only showed inhibition of FLS proinflammatory ability, but also showed that caspase-8 levels, a marker of ferroptosis, could be reduced (Zheng Q. et al., 2023). In addition to natural medicines, drugs targeting FLS modulation have now been developed and proven effective. Cathepsin B is a protease involved in joint injury and is highly expressed in articular cartilage in RA. Its inhibitor CA-074Me inhibited FLS proliferation and promoted FLS ferroptosis (Luo et al., 2024). Sulfasalazine, as a treatment for AS, has been shown to promote ferroptosis in FLS in RA (Zhao et al., 2024). Several differential gene analyses yielded genes involved in regulating ferroptosis in FLS. Several differential gene analyses yielded genes involved in regulating ferroptosis in FLS. Wang et al. analyzed eight ferroptosis genes associated with RA, of which TIMP Metallopeptidase Inhibitor 1 was significantly expressed in FLS (Wang et al., 2024b). Jing et al. identified SLC2A3 as highly expressed in FLS by bioinformatics methods and machine learning algorithms and experimentally verified that FLS treated with RSL3 exhibited SLC2A3 downregulation and underwent ferroptosis (Xiang et al., 2023). Therefore, it is well documented to treat differential genes. Nuclear Receptor Coactivator 4 (NCOA4) mediates LPS-induced ferroptosis in FLS and targeting NCOA4 may be an effective strategy for the treatment of RA (Wang Y. et al., 2024).

In addition to targeting FLS, ways to interfere with ferroptosis for RA have also been mostly reported. An injectable gel composed of folic acid-functionalized polydopamine and leonurine (Leon) inhibits joint inflammation caused by macrophages and protects cartilage from ferroptosis (Lv et al., 2024). It is a novel way to treat RA by carrying Fe_3_O_4_ and sulfasalazine by using macrophages as carriers (Ruan et al., 2024). In this experiment, macrophage carriers could be transported to sites of RA inflammation guided by inflammatory factors and under near-infrared light irradiation, Fe_3_O_4_ converted light energy to heat energy. This synergistic effect, instead, predisposes inflammatory cells and proliferating synovium to ferroptosis, thereby achieving the effect of treating RA.

### 5.5 Treatment strategies for OP

BMSCs are an important source of OBs and OCs differentiation is influenced by RANKL. When OP develops, OBs differentiation is inhibited, and OCs differentiation is enhanced. Targeting BMSCs and RANKL is therefore a way to treat OP. Ferroptosis has been mostly reported to affect BMSCs and RANKL, and OP based on ferroptosis is a hot area of current research.

As a naturally occurring phenolic chemical, Picein enhances BMSCs’ capacity for osteogenic differentiation while reducing oxidative stress caused by erastin via the Nrf2/HO-1/GPX4 pathway (Huang et al., 2024a). Overexpression of Crystallin Alpha B (CRYAB) increased OCN and Runx2 expression and increased ALP activity in BMSCs. Further experiments verified that CRYAB could interact with FTH1, inhibit ferroptosis of BMSCs, and promote osteogenic differentiation (Tian et al., 2024). BMSCs induced by high glucose and high fat environments exhibited bone degradation and ferroptosis, but this was reversed by poliumoside. When poliumoside was used in the T2DOP mouse model, increased bone mineral density and GPX4 expression were observed in the distal femur of mice, which also confirmed its effectiveness with cellular experiments (Xu et al., 2024). Based on a DNA tetrahedral nanoparticle involved in curcumin, tFNA-Cur, could inhibit ferroptosis in BMSCs and promote osteogenic differentiation in diabetic environment through Nrf2/GPX4 pathway (Li et al., 2024d). Ebselen is a selenium-containing organic drug molecule that can act as a mimetic of GPX. The experiment verified that Ebselen improved the ferroptosis and osteogenic differentiation inhibition status of BMSCs induced by LPS (Huang Z. et al., 2023). Engeletin, as an endogenous antioxidant, can promote osteogenic differentiation of BMSCs and upregulate osteogenesis-related proteins, which has achieved the effect of counteracting ferroptosis (Huang L. et al., 2023). Tocopherol as an antioxidant can reduce oxidative stress in BMSCs, promote osteogenesis-related protein expression, and inhibit ferroptosis in BMSCs (Lan et al., 2022b). Vitamin K2 has been used clinically to prevent OP. In cell experiments, vitamin K2 reversed ferroptosis and upregulated osteogenic marker expression in BMSCs under high glucose conditions (Jin et al., 2023).

In addition, novel medical devices have been developed to target the inhibition of osteogenic differentiation caused by ferroptosis. Targeted regulation of BMSCs metabolic status is a critical point to promote osteoblast differentiation. Therapeutic protocols targeting BMSCs metabolism were therefore designed and experimentally confirmed. Yang designed a titanium implant coated with caffeic acid and DFO. Titanium implants were implanted into the femoral epiphysis of OVX rats, and it was observed that titanium implants promoted new bone formation after 1 month. Its mechanism of action is to reduce the lipid peroxidation level of BMSCs by activating the KEAP1/NRF2/HMOX1 pathway and to activate and promote the SLC7A11/GSH/GPX4 axis to inhibit BMSCs ferroptosis (Yang Y. et al., 2024). Bone cement is commonly used to treat OP fractures. However, its cytotoxic properties make it likely to have an impact on osteogenic differentiation. A composite PDT-TCP-SE based on polylactide-based copolymer (PDT), β-tricalcium phosphate (β-TCP), and selenium nanoparticles (SeNPs) was developed to replace the application of traditional bone cements. It was found that PDT-TCP-SE could protect BMSCs from erastin-induced ferroptosis through Sirt1/Nrf2/GPX4 antioxidant pathway and had the effect of regulating new osteogenesis at OP fracture site (Huang et al., 2024b).

Saikosaponin A, a component of the natural medicine *Bupleurum falcatum*, can inhibit RANKL-induced OCs production and inhibit the Nrf2/SCL7A11/GPX4 axis to promote ferroptosis in OCs (Li TQ. et al., 2024). Zoledronic acid is a bisphosphonate that blocks ferrostatin-1 ‘s ability to induce osteoclast death. Moreover, the expression of ferroptosis-related expression in OCs treated with zoledronic acid was significantly increased, and these results indicated that zoledronic acid could puncture osteoclast ferroptosis (Qu et al., 2021). Further studies with zoledronic acid have found that it is through inhibition of the RANKL signaling pathway that OCs production is inhibited and bone loss is relieved (Wang B. et al., 2022). Artemisinin has been shown to downregulate RANKL-induced differentiation of OCs and has been used in place of treating bone loss caused by OCs. Because of the high iron content in OCs, the mechanism of action of artemisinin was identified as possibly associated with ferroptosis (Zhang, 2020).

Osteocyte metabolism affects OP caused by ferroptosis is rarely reported, however, as the most abundant cells in bone, regulating osteocyte metabolism is undoubtedly a worthwhile attempt. Activation of Activating Transcription Factor 2 (ATF2) has been found to induce ferroptosis in osteocytes, a phenomenon that is inextricably linked to age-related bone loss. AFT2 expression was suppressed and OP progression was slowed by administration of JY-2, a novel Forkhead Box O1 inhibitor (Yin et al., 2024). Eldecalcitol is an orally active vitamin D analogue (Sanford and McCormack, 2011). Eldecalcitol showed protection against bone in OVX mice induced by D-galactose. Further cell experiments confirmed that Eldecalcitol alleviated D-galactose-triggered ferroptosis, inhibited lipid peroxide accumulation, and enhanced GPX4 expression in MLO-Y4 cells (Fu et al., 2024).

In conclusion, ferroptosis can accelerate OP progression by affecting the osteogenic differentiation of BMSCs, target inhibition of ferroptosis in BMSCs, or promote ferroptosis in OCs induced by RANKL, which is a strategy worth applying for the treatment of OP.

### 5.6 Treatment strategies for OS

The treatment regimen of OS is consistent with conventional cancer treatment, that is, surgery, radiotherapy, chemotherapy and other modalities. However, due to its high incidence of lung metastasis, drug resistance and other characteristics, resulting in OS treatment effect is unsatisfactory. Induction of ferroptosis in tumor cells is currently the focus of treatment options, which also brings new perspectives for the treatment of OS. Multiple agents giving mechanisms of ferroptosis have been demonstrated to inhibit OS progression. EF24, an analog of curcumin, induced osteosarcoma cell death, the outcome of which was reversed by ferrostatin-1. In-depth studies have confirmed that it can increase MDA levels, ROS levels and increase intracellular iron content. HMOX1 expression was upregulated in a dose-dependent manner and promoted ferroptosis in osteosarcoma (Lin H. et al., 2021). The combination of ursolic acid and cisplatin induced intracellular overload of Fe^3+^, resulting in ferroptosis in osteosarcoma cells (Tang et al., 2021). Tipazamine can inhibit the expression of GPX4 and SLC7A11 under hypoxia and thus induce ferroptosis (Shi et al., 2021). Sulfasalazine and miR-1287-5p mimics could inhibit GPX4 to promote ferroptosis in osteosarcoma cells (Xu Z. et al., 2021; Liu J. et al., 2022). Bavachin, as a flavonoid compound, can promote ferroptosis in osteosarcoma cells by up-regulating p53, and down-regulating SLC7A11 and GPX4 (Luo et al., 2021). Several novel nanomedicines also exert a role in promoting ferroptosis in OS cells (Fu et al., 2021; Wang Y. et al., 2022). Ferroptosis is also involved in reducing drug resistance in osteosarcoma. miR-1287-5p rendered osteosarcoma cells more sensitive to cisplatin (Xu Z. et al., 2021). The same result was found in osteosarcoma cells after inhibition of Lysine Demethylase 4A expression (Chen M. et al., 2021). Combination of erastin, RSL3 and STAT3 inhibitors also increased sensitivity to cisplatin (Liu and Wang, 2019).

### 5.7 Treatment strategies for IVDD

NP cell reduction or death is the main cause of IVDD. Several studies have now been directed at inhibiting NP ferroptosis to maintain its normal physiological state. Shu et al. determined that Tinoridine could rescue RSL3-induced ferroptosis in NP cells by increasing Nrf2 expression by screening nonsteroidal anti-inflammatory drugs (Yang S. et al., 2024). Fisetin is involved in the regulation of the Nrf2/HO-1 pathway, thereby inhibiting NP ferroptosis (Li C. et al., 2024). Hesperidin can enhance Nrf2 expression and inhibit NF-κB, thereby alleviating ferroptosis resulting from oxidative stress in NP (Zhu et al., 2023b). HIF-1α promotes translation of SLC7A11 and reduces NP ferroptosis under hypoxia by inducing expression of the m6A reading protein YTHDF1 (Lu et al., 2024). DNA methyltransferase inhibitors prevented puncture-induced IVDD and protected NP from ferroptosis (Chen J. et al., 2024). *In vivo* experiments confirmed that targeting the miR-874-3p/ATF3 axis could modulate NP ferroptosis and is an effective way to treat IVDD (Wang X. et al., 2024). Circ-STC2 is a critical circRNAs involved in IVDD (Chang et al., 2021). In cell experiments against Circ-STC2, Circ-STC2 was found to be highly expressed in IVDD tissues. Knockdown of Circ-STC2 promoted NP cell viability and prevented from suffering ferroptosis (Xiong et al., 2023). A hydrogel containing SLC7A11-modRNA inhibited ferroptosis in NP cells by local injection, and the rate of SLC7A11-modRNA release positively correlated with IVDD severity (Gao et al., 2024).

## 6 Conclusion

Ferroptosis, a programmed cell death that is significantly different from other modes of cell death, is currently the focus of research in the field of disease mechanisms. The mechanism of ferroptosis mainly involves lipid peroxidation, iron accumulation and antioxidant system, which makes it independent of the mode of cell death. Because of this, diseases associated with ferroptosis can be found in various tissues and organs of the human body. The occurrence of bone disease is closely related to the physiological status of bone. Abnormal physiological metabolism of cells in bone tissue can cause various types of bone diseases. Ferroptosis can be involved in any ring of metabolism in cells. Multiple core regulators involved in ferroptosis, such as GSH, GPX4, System X_c_^−^, Nrf2, and ROS, have also been demonstrated to be involved in the development of skeletal diseases.

However, it is undeniable that although iron death has been increasingly studied in the field of orthopedics, it still faces non-negligible challenges and limitations in clinical application. Iron death may have different effects on osteoblasts and osteoclasts, either protecting bone health or exacerbating bone loss under certain conditions. This dual effect makes it difficult to precisely regulate the degree and balance of ferroptosis in treatment. Current studies on iron death mostly focus on tumor cells and other non-skeletal tissues, and there is still a lack of in-depth studies targeting skeletal-related cells such as osteoblasts, osteoclasts, and chondrocytes. Susceptibility to ferroptosis and mechanisms may differ between cell types. Accumulation of iron in skeletal cells may lead to cellular dysfunction or even induce apoptosis, which is not conducive to bone health. In addition, regulation of iron metabolism by exogenous means may lead to altered systemic iron load and trigger adverse effects in other organs and tissues, such as iron accumulation in the liver and heart. In addition, the response to ferroptosis regulation may vary widely between individual patients, and different bone disease types (e.g., osteoarthritis *versus* osteoporosis) and disease duration stages respond differently to iron death interventions. Therefore, how to develop a precise ferroptosis regulation program according to the individual condition of patients is a major challenge for future application.

In this paper, we try to summarize the current research progress of ferroptosis in detail by elaborating the development process of ferroptosis, the intrinsic mechanism, and the relationship with other cell death modes. The mechanism of ferroptosis in many bone diseases is also listed. Finally, the current research on the treatment of bone diseases by affecting ferroptosis is summarized. Because ferroptosis involves a wide range of fields and complex mechanisms, research on the effects of ferroptosis on bone disease remains to be continued. We hope to provide efficient and safe treatment modalities and preventive strategies for clinical as well as scientific research through this paper and further research in the future.
